# Geostatistical Modelling and Web‐Based Mapping of Malaria Risks Among Children Under Five Years in Nigeria: Evidence From the 2021 Nigeria Malaria Indicator Survey

**DOI:** 10.1002/hsr2.73006

**Published:** 2026-08-05

**Authors:** Justice Moses K. Aheto, Bakare Emmanuel Afolabi, Dolapo O. Oniyelu, Deborah O. Daniel, Steven I. Ikediashi, Oluwaseun A. Mogbojuri, Ronke D. Olorunfemi, Sodiq A. Orogun, Afeez Abidemi, Idowu I. Olasupo, Samuel A. Osikoya, Samuel A. Adeyemi, Hapiness O. Ismail, Isaac Bakare, Samson O. Olagbami, Dolapo A. Bakare, Baiyeri Samuel, Temitayo V. Irewole, Oluwafunmilayo O. Olapade, Oluwafolakemi Odunola, Oghenekevwe R. Ajewole, Joshua P. Ojo, Segun Oyedeji, Wisdom Takramah

**Affiliations:** ^1^ Department of Biostatistics, School of Public Health, College of Health Sciences University of Ghana Accra Ghana; ^2^ WorldPop, School of Geography and Environmental Science University of Southampton Southampton UK; ^3^ College of Public Health University of South Florida Tampa Florida USA; ^4^ Department of Mathematics Federal University Oye Ekiti Oye Nigeria

**Keywords:** Children under‐five, geospatial modelling, integrated nested Laplace approximation, malaria, Nigeria, sub‐Saharan Africa, under‐five malaria, web‐based mapping

## Abstract

**Background:**

Malaria is a critical public health concern in Nigeria with the country bearing an uneven burden of the disease. In Nigeria, malaria is one of the main causes of child mortality and despite all efforts to reduce malaria mortality rates, the disease remains a major concern, notably among children under‐fives. There is a paucity of data on more localized predictive malaria risk geospatial maps to inform control and elimination strategies amidst limited public health resources in this setting. This modelling study therefore sought to understand, predict and map malaria risk in the presence of environmental factors in Nigeria.

**Methods:**

This study utilized data from the 2021 Nigeria Malaria Indicator Survey (NMIS), conducted under the Demographic and Health Surveys (DHS) Program. The 2021 NMIS marks the third malaria indicator survey carried out in Nigeria, following previous surveys in 2010 and 2015. The outcome variable of interest is the number of individuals in each sampled cluster who tested positive on the rapid diagnostic test (RDT). This study investigated spatial risk factors for malaria prevalence in Nigeria through geostatistical modelling approaches. The implementation of the models was carried out with the integrated nested Laplace approximation (INLA) method via the stochastic partial differential equation (SPDE) approach in R‐INLA.

**Results:**

The study identified aridity (log‐odds = −0.0400, 95% CrI = −0.0610, −0.0190) and enhanced vegetation index (log‐odds = 9.3930, 95% CrI = 7.4220, 11.3760) as significant predictors of under‐five malaria risk. The fitted Bayesian geospatial spatial model with covariates predicted malaria prevalence of 24.9% with a range of 0.5% to 74.2%. The predicted malaria prevalence was highest in parts of Zamfara (prevalence > 70%) and Kebbi (prevalence > 60%) States.

**Conclusion:**

These findings are of the utmost significance for policymakers involved in malaria control and elimination efforts. They provide evidence‐based information that can guide resource mobilization and targeting, intervention design, and monitoring strategies. The identification of environmental predictors like aridity and enhanced vegetation index suggests the need for location‐specific interventions according to environmental determinants of malaria transmission.

**Abbreviations**
CM: Case Management
DHS: Demographic and Health Surveys
DIC: Deviance Information Criterion
EA: Enumeration Area
EVI: Enhanced Vegetation Index
INLA: Integrated Nested Laplace Approximation
IPTp: Intermittent Preventive Treatment of Malaria During Pregnancy
ITNs: Insecticide‐Treated Nets
LGA: Local Government Authority
LLINs: Long‐Lasting Insecticides Treated Nets
MCMC: Markov Chain Monte Carlo
MIS: Malaria Indicators Survey
NBS: National Bureau of Statistics
NDHS: Nigeria Demographic and Health Survey
NDVI: Normalized Difference Vegetation Index.
NMEP: National Malaria Elimination Programme
NMIS: Nigeria Malaria Indicator Survey
NMSPs: Nigeria Malaria Strategic Plans
NPC: National Population Commission
PHC: Population and Housing Census
RDT: Rapid Diagnostic Test
SE: Standard Error
SMC: Seasonal Malaria Chemoprevention
SPDE: Stochastic Partial Differential Equation
WAIC: Watanabe‐Akaike Information Criterion

## 1 Background

Plasmodium‐infected female Anopheles mosquitoes are responsible for the transmission of malaria, with the greatest disease burden occurring among children under 5 years of age [1]. Five *Plasmodium* species cause malaria in humans, *with P. falciparum* being responsible for the most severe and life‐threatening infections, particularly among children under 5 years and pregnant women [1, 2]. In 2022, approximately 249 million malaria cases and 608,000 deaths were reported globally, with about 94% of the cases occurring in Sub‐Saharan Africa [3].

Malaria is a critical public health concern in Nigeria with the country bearing an uneven burden of the disease. In Nigeria, malaria is one of the main causes of child mortality and despite all efforts to reduce malaria mortality rates, the disease remains a major concern, notably among children below the age of five. In 2021, Nigeria had an estimated 68 million cases and 194,000 deaths due to the disease [3]. Nigeria has the highest burden of malaria, which accounts for nearly 27% globally [3]. The threat of transmission exists throughout the year in every part of the country with the highest incidence in the northern parts of the country [3, 4].

Nigeria has executed four National Malaria Strategic Plans (NMSPs) and is currently carrying out the fifth plan, spanning from 2021 to 2025 [5]. The current fifth plan targets a parasite prevalence of less than 10% and aims to decrease malaria‐related mortality to fewer than 50 deaths per 1,000 live births by 2025 [5]. To achieve its 2025 target, the National Malaria Elimination Programme (NMEP) has rolled out several interventions, such as long‐lasting insecticides treated nets (LLINs), intermittent preventive treatment of malaria during pregnancy (IPTp), seasonal malaria chemoprevention (SMC) and case management (CM) [6].

In 2021, Nigeria conducted the third (the first and second were in 2010 and 2015 respectively) malaria indicator survey (MIS) to provide key information on malaria indicators at the national, zonal, and state levels and reported malaria prevalence of 39.6% based on Rapid Diagnostic Test (RDT) results and 22.3% based on Microscopy test results [7]. The 2021 survey, which was a follow‐up to the 2015 survey was jointly implemented by the NMEP, the National Population Commission (NPC), and the National Bureau of Statistics (NBS), with technical support from ICF International [7]. The 2021 survey covered a total of 568 clusters, an improvement on the 2010 and 2015 surveys which covered a total of 240 and 330 clusters respectively [7].

Several research works have been carried out to evaluate the prevalence of malaria and socio‐demographic risk factors in Nigeria [8, 9, 10, 11]. A previous study employed the 2010 Nigeria Malaria Indicator Survey (NMIS) data to identify factors associated with malaria risk in Nigeria [12]. They generated a risk map of malaria among under‐five children and utilised Bayesian geostatistical models fitted via Markov Chain Monte Carlo (MCMC) to estimate parameters and make predictions. Their result shows that the most important drivers of malaria risk in Nigeria are rainfall and the Normalized Difference Vegetation Index (NDVI).

Based on the 2015 NMIS data, a previous study investigated the correlations between malaria prevalence in under‐five children and socioeconomic, demographic, and geographic factors in Nigeria [9]. By using Kriging, predictive risk factor maps were developed based on the survey data, to identify high‐risk regions. They observed that malaria prevalence was more rampant among under‐five children living in low‐income households which agrees with previous studies that socioeconomic factors such as poverty strongly influence the vulnerability of children under‐five to malaria infection.

Another study presented a spatiotemporal analysis of malaria prevalence and investigated environmental disparities in Nigeria using the 2021 NMIS data [4]. The study revealed that the enhanced vegetation index, annual land surface temperature, precipitation, insecticide‐treated nets (ITNs) coverage, and mean temperature are key factors influencing malaria prevalence.

The combination of geostatistical modeling and interactive web‐based mapping offers a more promising approach to addressing the malaria burden in Nigeria, leveraging the insights from the 2021 MIS to guide targeted and effective interventions.

## 2 Methods

### 2.1 Study Setting and Population

Nigeria is located in West Africa and covers a total area of 923,769 square kilometres (356,669 sq mi) [7]. It lies between latitude 40N and 140N of the equator and between longitude 30E and 150E. The country has a population of more than 230 million and shares borders with the Republic of Benin to the west, Chad and Cameroon to the east, the Gulf of Guinea of the Atlantic Ocean to the south, and Niger to the north. Currently, Nigeria has 36 states and the Federal Capital Territory [7].

Data on malaria cases among children under 5 years extracted from the 2021 NMIS of the DHS program was used in this study. The 2021 NMIS is the third malaria indicator survey conducted in Nigeria, with the first in 2010 and the second in 2015. The survey provides insights into the impact of the interventions implemented so far and possible revisions of strategies, the sample size for the 2021 NMIS was much larger than in previous surveys, with a total of 568 clusters covered across the country (195 in urban areas and 373 in rural areas) used to inform strategic planning and evaluation of the Nigeria Malaria Control Programme [7]. Computer‐assisted personal interviewing (CAPI) was employed to collect the data.

In the survey, information on malaria prevention, treatment, and prevalence is collected. The data is freely available online at DHS MEASURE Program website (MEASURE DHS). Parents or guardians' consent were sought for children aged 6–59 months who were tested for anaemia and malaria infection. The study used a biomarker questionnaire to record the results of the anaemia and malaria testing of the children aged 6–59 months. Also, this study used data on under‐five children from the biomarker datasets which have malaria RDT results on 11,564 under‐five children residing in 568 geographical locations (clusters). Detailed description of the survey methods employed in the 2021 NMIS is available elsewhere [7].

The sampling framework/design used for the 2021 NMIS was the cartographic frame of the National Population Commission (NPC) for the proposed 2023 Population and Housing Census (PHC). Administratively, Nigeria is divided into states. Each state is subdivided into LGAs, each LGA is divided into wards, and wards are further subdivided into localities. In addition to these administrative units, localities are subdivided into convenient areas called census enumeration areas (EAs). The primary sampling unit (PSU), referred to as a cluster for the 2021 NMIS, was defined on the basis of the EAs from the census frame for the proposed 2023 PHC [7].

The sample design for the 2021 NMIS was a stratified sample selected in two stages. Stratification was achieved by separating each of the 36 states and the Federal Capital Territory into urban and rural areas [7]. In total, there were 73 sampling strata and samples were selected independently in every stratum through a two‐stage selection. Implicit stratification was achieved at each of the lower administrative levels by sorting the sampling frame before sample selection according to administrative order and by using probability proportional to size selection in the first stage's sampling. In the first stage, 568 EAs were selected with probability proportional to EA size. EA size is the number of households residing in the EA [7]. A household listing operation was carried out in all selected EAs, and the resulting lists of households served as a sampling frame for the selection of households in the second stage. In the second stage's selection, a fixed number of 25 households were selected in every cluster via equal probability systematic sampling [7].

According to Nigeria Demographic and Health Survey (NDHS), the average number of women aged 15–49 per household was 1.04, and the average number of children aged 5–59 months per household was 0.86. The household completion rate was 97%, the women's response rate was 99%, and the children's response rate was about 96% for both RDT and microscopy [7].

### 2.2 Outcome Variable

The outcome variable of interest is the number of individuals in each sampled cluster who tested positive on the rapid diagnostic test (RDT) kit. Using the Biomarker Questionnaire, individuals had their malaria rapid diagnostic testing results recorded. With the aid of the Nigeria‐approved SD BIOLINE Malaria Ag P. f. (HRP‐II)TM RDT, blood samples for biomarker testing were taken via finger or heel pricks and were tested immediately.

This qualitative test detects the histidine‐rich protein II antigen of Plasmodium falciparum in human whole blood. Malaria in Nigeria is mostly caused by the P. falciparum parasite, which is transmitted by the Anopheles mosquito. The diagnostic test is included with a single‐use, standard‐package disposable sample applicator. A small volume of blood is collected on the applicator and put into the testing device's well. According to the guidelines provided by the manufacturers, all biomarker specialists received training on how to conduct RDTs in the field. Within 15 min, the RDT findings were available. They may be classified as positive or negative, with weak test lines being interpreted as positive [7].

### 2.3 Independent Variables

The primary objective of this research is to predict and map the risk of malaria in Nigeria while simultaneously adjusting for critical climatic and environmental factors that can help explain possible geographical variations in malaria risk across Nigeria. These factors include night‐land surface temperature, nightlights composite, enhanced vegetation index (from least to most), aridity (from most arid to most wet), precipitation, wet Days as well as demographic factors like under‐five population. The methods and procedures used in this work were in accordance with the geospatial covariate datasets manual [13].

### 2.4 Geospatial Analysis

#### 2.4.1 Model Formulation

A Bayesian geostatistical model was applied to the data to estimate malaria prevalence and identify spatial risk factors among children under 5 years of age. Let $Y_{i}$denotes the total number of malaria cases out of the total population $N_{i}$ sampled per geographical cluster. Conditional on the true prevalence P($s_{i}$) at location $s_{i}$, the number of malaria cases among the sampled population is assumed to follow a binomial distribution:

Y∣P(s)∼Binomial(n,P(s)),logit(P(s))=α+d(s)⊤β+S(s),

where α is the intercept term, assigned a Gaussian prior with mean 0 and precision 0.001; d(s)is the vector of observed spatial covariates at location sassociated with the outcome; βis the vector of spatial regression coefficients for the covariates, assigned a Gaussian prior with mean 0 and precision 0.001; and S(s)represents the spatial random effect, assumed to follow a zero‐mean Gaussian process with variance $\sigma^{2}$and a specified correlation function.

$$\rho (u)=\mathrm{corr}\left\{S\left(s_{i}\right),S(s_{j})\right\},$$

where u denotes the Euclidean distance between the locations $s_{i}$ and $s_{j}$. We used Gamma (1,0·00005) as the priors for our precision parameters associated with the scaled models. We further chose to use the non‐informative technique when determining our priors because there was no reliable existing information about the parameters of our model. Generally, the selected priors are used as default priors in the R‐INLA package [14, 15]. Diggle and Ribeiro outlined several parametric families for ρ(u) [16]. In this analysis, we employ the Matérn class of covariance function [17, 18], which is given by

$$\mathrm{Cov}\left(Z\left(s_{i}\right),Z\left(s_{j}\right)\right)=\frac{\sigma^{2}}{2^{\upsilon -1}\Gamma (\upsilon )}{\left(\kappa \text{||}x_{i}-x_{j}\text{||}\right)}^{\upsilon }K_{\upsilon }(\kappa \text{||}{\mathrm{xs}}_{i}-s_{j}\text{||})$$

The modified Bessel function of second kind and order v > 0 is denoted by $K_{\upsilon }$ (.), while the variance is represented by σ^2^. S(s) is (v–1) times mean‐square differentiable, and its smoothness is determined by the shape parameter ν. A value of κ > 0 corresponds to the practical range ρ = √8v/κ, which is the distance at which the spatial correlation approaches 0.1.

Implementation of the model was carried out using the integrated nested Laplace approximation (INLA) approach [19, 20] with the stochastic partial differential equation (SPDE) strategy [21]. It was necessary to create the mesh for the SPDE strategy since, unlike areal data, our data are point data without specified neighbors. The study accounted for the cluster displacement which is an inherent characteristic of the Demographic and Health Survey data. A detailed explanation of the procedures used here can be found in previous studies [18, 20, 22]. Several spatial and non‐spatial models were set up. Next, we used the Watanabe‐Akaike information criterion (WAIC) and the deviance information criterion (DIC) to select the model that fits the data better among the competing models. For our predicted malaria prevalence surfaces, we consistently generated 95% Bayesian credible intervals for the entire country of Nigeria to quantify the uncertainty around our estimates. Multicollinearity was examined using variance inflation factor (VIF) and VIF value< 10 considered acceptable (i.e., no noticeable evidence multicollinearity). R‐INLA package [14] was used for the implementation of all the analyses.

#### 2.4.2 Model Validation

It is essential to analyze how well the predictive model performs, particularly when new data is available. The 10 folds cross‐validation was used in this work to evaluate the predictive performance of the model under out‐of‐sample procedures. To make the partition reproducible, the data was first split into training and validation sets, with a seed of 123. 25% of the samples were used for testing after the model had been trained on 75% of them. The study was allowed to evaluate the predictive performance of the model by estimating correlation between the observed and the predicted malaria prevalence.

#### 2.4.3 Interactive Web‐Based Mapping of the Predicted Malaria Prevalence

To better visualize and identify higher‐risk communities for immediate intervention and additional research in this setting where universal intervention is nearly impossible due to limited public health resources, the study produced interactive web‐based maps for predicted malaria prevalence. This was done to support policymakers with readily available quality data for targeted policy and intervention strategies, especially for malaria surveillance and elimination amidst limited public health resources in these settings. With the aid of the leaflet, and sp packages in R version 4.4.1 and RStudio [23, 24], we developed the interactive web‐based predicted malaria prevalence maps.

### 2.5 Ethical Approval

This study was based on a publicly available dataset from the MEASURE DHS program. No ethical approval was needed since it did not directly involve contact between the authors and the individuals interviewed. However, the survey protocol was reviewed and approved by the National Health Research Ethics Committee of Nigeria (NHREC) and the ICF Institutional Review Board [7]. The risk and benefits of participation in the survey were explained to respondents, including informed consent for the interview or blood collection, and informed consent was sought from all respondents. Also, the data used for this study has no contact details of the interviewed participants and households, and permission was sought from and given by the MEASURE DHS program for this study via the online portal http://www.dhsprogram.com.

## 3 Results

### 3.1 Model Selection

Malaria prevalence was predicted by comparing closely matched good models and arriving at the best model, employing the Watanabe‐Akaike information criterion (WAIC) [25, 26]. After implementing the seven competing models, Model 7 had the lowest WAIC value (WAIC=3034.29), and thus most preferred model for the study. This model included covariates such as Aridity, Enhanced Vegetation Index, Nightlights Composite, and Night Land Surface Temperature, as presented in Table 1. The results based on Model 7 are thus investigated as presented in Table 2.

**Table 1:** Model selection parameters for all models explored.

Parameters	WAIC	DIC
**Full non‐spatial model**		
Model 1: Enhanced Vegetation Index, Nightlights Composite, Wet Days, Night Land Surface Temperature	4763.14	4757.91
Model 2: Aridity, Enhanced Vegetation Index, Nightlights Composite, Precipitation, Wet Days, U5 Population	4154.6	4116.05
Model 3: Aridity, Enhanced Vegetation Index, Nightlights Composite, and Night Land Surface Temperature	4741.63	4277.2
**Spatial models**		
Model 4: Null spatial model	3170.3	2955.46
Model 5: Enhanced Vegetation Index, Nightlights Composite, Wet Days	3042.36	2855.65
Model 6: Aridity, Enhanced Vegetation Index, Nightlight Composite, and Night Land Surface Temperature, Wet Days and U5 Population	3043.8	2856.42
Model 7: Aridity, Enhanced Vegetation Index, Nightlights Composite, and Night Land Surface Temperature	3034.29	2849.78

**Table 2:** Predictors of malaria prevalence in the Bayesian spatial models.

Parameter	Null spatial model	Full spatial model
	Mean log odds (95% Cr.I)	Mean log odds (95% Cr.I)
**Fixed effect**		
Intercept	−0.46 (−0.65, −0.28)	0.12 (−1.30, 1.54)
Aridity		−0.04 (−0.06, −0.02)
Enhanced vegetation index		9.39 (7.42, 11.38)
Nightlights composite		−0.01 (−0.02, 0.01)
Night land surface temperature		−0.11 (−0.25, 0.02)
**Random effect**		
Kappa (k)	6.62 (4.07, 9.62)	3.19 (2.24, 4.22)
Spatial variance (σ^2)	1.82 (1.16 2.57)	1.46 (1.09, 1.85)
Range (r)	0.45 (0.27, 0.63)	0.91 (0.64, 1.20)

*Note:* Cr.I: Bayesian Credible Interval.

### 3.2 Predictors of Malaria Prevalence From the Bayesian Spatial Models

Table 2 presents the results from Models 4 and 7 to allow comparison between the null spatial model (Model 4) and the preferred spatial model with covariates (Model 7), but the interpretations presented in this paper are based on the spatial model with covariates (Model 7). Aridity shows statistical significance with log‐odds of −0.0400 and a 95% credible interval of −0.0610 to −0.0190. The enhanced vegetation index is significantly associated with malaria episodes with log‐odds of 9.3930 and the 95% credible interval of 7.4220 to 11.3760. The log‐odds for the Nightlights Composite was −0.0060 with a 95% credible interval of −0.0180 to 0.0070 and the Night Land Surface Temperature with log‐odds of −0.1140 with a 95% credible interval of −0.2450 to 0.0150 albeit insignificant. Additionally, we estimated the spatial variance ($\sigma^{2}$) to be 1.46, with a 95% credible interval from 1.09 to 1.85. The estimated range is 0.91, with a 95% credible interval from 0.64 to 1.20, and the kappa (κ) is 3.19, with a 95% credible interval from 2.24 to 4.22.

### 3.3 Geospatial Analysis and Interactive Web‐Based Mapping

**Figure 1:**
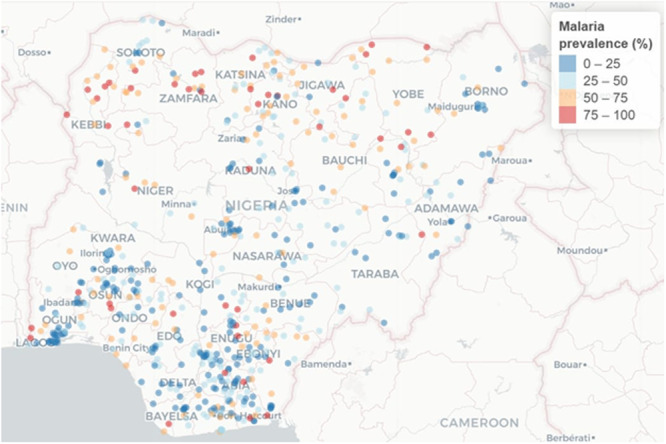
The empirical (observed) prevalence of malaria among under‐five children in the Nigerian study areas in 2021. The circles indicate the centroid of the sampled cluster study locations.

Data on 10717 children located in 567 spatially indexed communities were evaluated for this study. The study's community locations (centroids of clusters) and the corresponding empirically observed malaria prevalence (coloured) in the sampled locations are displayed in Figure 1. Communities with blue‐highlighted circles had a malaria prevalence of 0%–25%, while communities with red highlighted circles had a malaria prevalence of 75%–100%.

**Figure 2:**
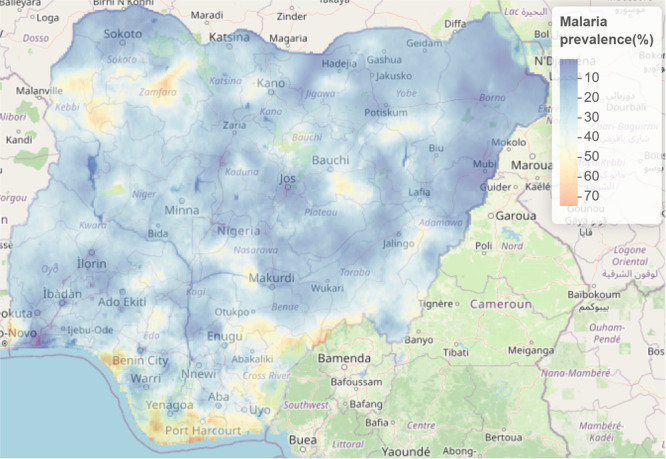
Malaria prevalence among under‐five children predicted in 2021 in Nigeria. The map is accessible online as an interactive web‐based version.

**Figure 3:**
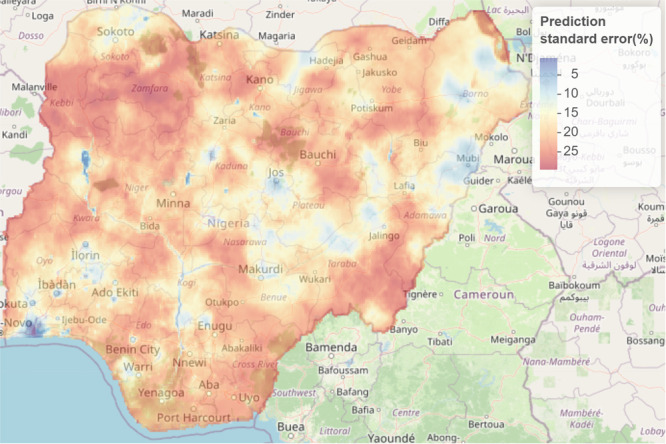
Level of uncertainty associated with the predicted malaria prevalence among under‐five children in 2021 in Nigeria.

The fitted Bayesian geospatial prediction model strongly predicts malaria prevalence spatially with a high correlation of 0.89. The study found substantial geographical differences in Nigeria's malaria prevalence across the country. The geospatial model with covariates predicted a prevalence of 24.9% (SE 14.8%) with a range of 0.5% to 74.2%. The predicted prevalence in parts of Kebbi (> 70%), Yobe (within the range of 60%–70%), and Zamfara (within the range of 50%–60%) states were noted to have the highest risk of under‐five malaria after applying the necessary covariate adjustments. These states are primarily located in the northern parts of the country, and states also having relatively high risk (range 50%–60%) include Bauchi, Jigawa, and Kano. Among the states that recorded a prevalence > 40%, two (2) were from the southern parts of the country (Ondo and Cross Rivers), and the other three (Katsina, Niger, and Sokoto) are still from the northern part of Nigeria. Some of the states that showed the lowest prevalence (< 20% (Figure 2) include Delta, Borno, Kwara, and Lagos. The interactive web‐based version of Figure 2 can be found in Supporting Information Figure S1. The standard errors (SEs) were presented in Figure 3 to quantify the uncertainty associated with our estimates presented in Figure 2. The estimated mean SEs is 14.8% with a range of 0.7% to 29.5%, suggesting a low level of uncertainty about the estimates and hence reliable estimates.

### 3.4 Comparing the Spatial Model With Covariates and the Spatial Model Without Covariates

**Figure 4:**
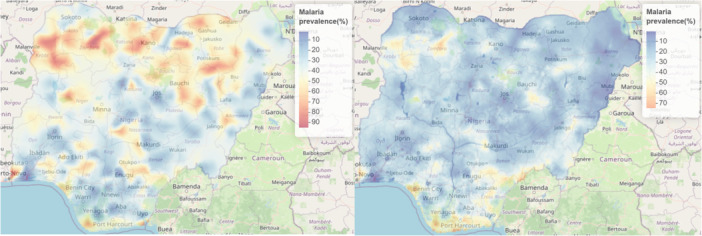
Predictive map for the spatial model without covariates (left panel) compared to those with covariates (right panel) for malaria prevalence in 2021 among under‐five children in Nigeria, from the Bayesian spatial models.

To facilitate understanding of the contribution of the spatial covariates to the malaria prediction, the study compared the predicted malaria prevalence for both the spatial model without geospatial covariates and spatial model with geospatial covariates. The spatial model without covariates predicted malaria prevalence of 40.5% (SE 12.5%) with a range of 2.17% to 94.7%, while the spatial model with covariates predicted a prevalence of 24.9% (SE 14.8%) with a range of 0.5% to 74.2%. Thus, including these spatial covariates helped explain some of the geographical differences in malaria prevalence across Nigeria, especially in the northern parts of the country (Figure 4).

**Figure 5:**
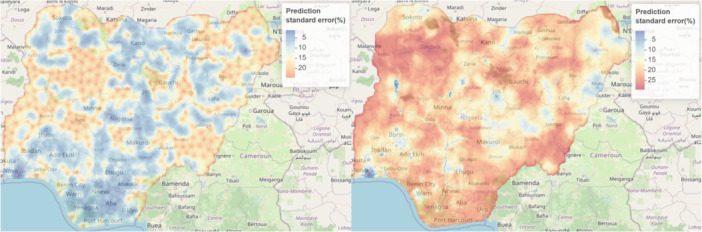
Comparing the level of uncertainty between maps for the spatial model without covariates (left panel) and the spatial model with covariates (right panel) from the Bayesian geospatial models for malaria prevalence in 2021 among under‐five children in Nigeria.

We reported the SEs for the predictions shown in Figure 4 to assess the level of precision of the estimates for the two models. We observed a slightly higher level of uncertainty (i.e., average precision) for the estimates in the spatial model with covariates compared to the spatial model without covariates (Figure 5). The mean SE associated with the spatial model with covariates was 14.8%, compared to 12.5% for the spatial model without covariates.

The study also evaluates the results between the predicted and observed malaria risk using the predictions obtained from the spatial model with covariates and found a significant correlation of 0.84, indicating a good agreement between the observed and predicted malaria risks in the study.

## 4 Discussion

### 4.1 Principal Findings

Controlling, preventing, and eliminating the problem of under‐five malaria risks is critical for the growth and development of children, the growth of national economies, and the survival of nations globally. The use of reliable and accurate data is essential for informing evidence‐based policies aimed at malaria elimination and prevention in Nigeria. The present study attempted to identify climatic and environmental factors that predict under‐five child malaria and to map the predicted malaria risk across both sampled and unsampled geographical locations in Nigeria. The malaria prevalence of 39.6% in children under 5 years as reported in the main survey [7] is relatively high compared to other countries in the subregion. For instance the malaria prevalence in children under 5 years in Ghana as reported in 2019 Malaria Indicators Survey (MIS) of the Demographic and Health Survey Program [27] was 23.0% and another study conducted in Ghana recorded 25% [18]. The pooled prevalence of under‐five malaria from systematic review and meta‐analysis carried out in Ethiopia was 22.03% [28]. This finding suggested that interventions may be failing to effectively reach the most vulnerable populations in Nigeria. In the present study, the predicted malaria prevalence is 24.9% (SE 14.8%) with a range of 0.5% to 74.2% after adjusting for the climatic and environmental factors in the geospatial model. Thus, the prevalence of malaria varies across Nigeria and a more localized interventions will probably be the most plausible measure to undertake to prevent and eliminate malaria in children aged 5 years.

### 4.2 Interpretation

The study showed that climatic and environmental factors such as aridity and enhanced vegetation index obtained from the fitted Bayesian spatial model were significantly associated with the prevalence of malaria in children aged < 5 years. The study identified aridity as a significant factor that reduces the risk of malaria in children under 5 years. This finding is consistent with what is known about malaria ecology where arid regions have less rainfall and fewer water bodies, reducing mosquito breeding grounds resulting in low transmission rate [29]. Regions characterized by low humidity and limited precipitation typically have lower population densities and more widely dispersed settlements, which in turn reduces the likelihood of mosquito‐human contact and consequently lowers malaria transmission potential. In contrast, a related study found that rapid urbanization commonly occurs in semi‐arid zones, leading to sharp increases in population density and potentially driving a shift toward human‐biting mosquito behavior in many African cities by 2050 [30], Enhanced vegetation index was another climatic factor identified in this study to have increased the likelihood of malaria risk in children under age five in Nigeria. The findings of this study were consistent with previous studies [29, 31]. This finding suggests that the Enhanced Vegetation Index (EVI) should be given significant consideration in malaria elimination efforts, as rising EVI values may serve as an early warning indicator for an increased risk of malaria among children under five. A strong positive association between the Enhanced Vegetation Index (EVI) and malaria among children under 5 years, accompanied by a large effect estimate, is biologically plausible because dense vegetation provides suitable habitats for Anopheles mosquitoes by increasing humidity and creating favourable breeding and resting environments. However, the magnitude of the estimated effect suggests that the relationship is unlikely to represent a simple, direct one‐to‐one increase in malaria risk with increasing EVI. Instead, it is more likely to reflect the scaling of the EVI variable together with the complex ecological pathways through which vegetation influences malaria transmission. Consequently, the estimated log‐odds should be interpreted with caution, as a one‐unit increase in EVI is rarely observed in practice and does not represent a realistic ecological change. Rather than indicating an implausibly large biological effect, the estimate primarily reflects the measurement scale of the predictor. Furthermore, areas with dense, mature vegetation represent distinct ecological and epidemiological settings that are characterised by higher humidity, favourable microclimatic conditions, and increased availability of mosquito breeding and resting sites, all of which can substantially enhance malaria transmission.

The observed malaria prevalence map for children under five revealed notable spatial heterogeneity, with particularly high prevalence in northern states such as Kebbi, Zamfara, Kano, Katsina, and Jigawa. A similar study also reported high prevalence of malaria in children under 5 years in the northern part of the country [31, 32]. Additionally, the fitted Bayesian geospatial prediction model effectively captured the spatial distribution of malaria risk. The results indicate that the inclusion of geospatial covariates contributed to explaining some of the geographic variations in the predicted malaria prevalence. After adjusting for these geospatial covariates, the highest predicted malaria prevalence in children under 5 years was observed in parts of Kebbi, Yobe, and Zamfara (50%–60%), all situated in the northern part of Nigeria. Other states in the northern part of Nigeria such as Bauchi, Jigawa, and Kano also exhibited relatively high prevalence rates. Among the states with prevalence rates exceeding 40%, two (2) were from the southern parts of the country (Ondo and Cross River), while the remaining three (3) Katsina, Niger, and Sokoto were from the northern part of the country. Conversely, the lowest prevalence rates were recorded in Delta, Borno, Kwara, and Lagos.

Spatial variation of malaria risk among children under 5 years was observed with the states in the northern part of the country bearing the most risk. Such a differential is likely to be explained by the interactions between climatic factors (vegetation cover, aridity), socio‐economic status, and availability of care. The covariate adjustment in the model allowed for more advanced understanding of malaria risk distribution, which showed that despite controlling for key predictors, some states primarily in the northwest and northeast are still transmission hotspots [32]. The presence of relatively high prevalence in southern states like Ondo and Cross River suggests localized risk factors such as high rainfall and high vegetation cover that facilitate vector breeding. On the other hand, decreased prevalence in urban or coastal areas like Lagos and Delta may reflect better access to health care, urbanization, and vector control. These results are critical for geographically targeted malaria interventions, especially in high‐transmission settings. The inclusion of the covariate explained some of the differences in malaria prevalence across Nigeria, especially in the northeastern and northwestern parts of Nigeria like Zamfara, Kano, Potiskum and Kebbi.

The study based on the 2021 Nigeria Malaria Indicator Survey (NMIS) offers several novel contributions that distinguish it from previous malaria research in Nigeria. Unlike earlier NMIS analyses, this study employed the integrated nested Laplace approximation (INLA) with the stochastic partial differential equation (SPDE) approach in R‐INLA. This method allows for more precise spatial modeling of malaria risk, capturing complex spatial dependencies and uncertainties that traditional models may overlook. The identification of aridity and the enhanced vegetation index (EVI) as significant climatic and environmental predictors of under‐five malaria risk provides novel evidence on how environmental conditions shape malaria transmission in Nigeria. Furthermore, the study highlights localized high‐burden hotspots in Zamfara and Kebbi States, offering granular insights that can guide geographically targeted malaria control strategies. By isolating risk factors specific to children under five, the study provides actionable insights for pediatric malaria control, a refinement over earlier NMIS studies that often aggregated data across age groups. These innovations make the study a valuable step forward in malaria epidemiology and control strategy design in Nigeria.

### 4.3 Strengths of the Study

The key strength of the study lies in the ability of the fitted model to borrow information from sampled geographical locations for the unsampled geographical locations in Nigeria for our spatial predictions and mapping for the whole of Nigeria in the presence of climatic and environmental factors while accounting for the cluster displacements. Another strength of the study lies in its representativeness and nationwide coverage, making it possible to generalize the findings to the population of children under‐fives in Nigeria and in similar populations elsewhere.

### 4.4 Limitation of the Data

Though this study has several strengths, there are some limitations which should be considered when interpreting the findings. The study lacks the ability to draw causative inference because it is cross‐sectional in nature. Furthermore, potential predictors like geographically indexed data on malaria policies and interventions, type of road network to the health facilities, distance to health facilities, and type of health facilities at the sampled and the unsampled locations were not available to be included in our Geospatial model to understand their contributions to malaria risks spatially in this population of children.

In geostatistical models with an explicit spatial field (SPDE) the spatial random effect may capture much of the average risk pattern. Depending on how the spatial field is the intercept can shift and become more uncertain because some of the overall level is explained partly by the random field rather than the intercept. Flat or uninformative priors on intercept and weak priors on precision of random effects can yield a wide posterior for the intercept. A wide intercept credible interval implies greater uncertainty in the model's estimate of the baseline log‐odds and therefore baseline prevalence, even if some covariate effects are estimated precisely.

The spatial field may be capturing some of the same spatial signal as EVI, both spatially structured, leading to instability in the EVI estimate. Flat priors and the spatial field can produce spuriously large posterior means.

## 5 Conclusion

This study highlights significant spatial disparities in malaria prevalence among children under five in Nigeria, with the highest burden concentrated in the northern part of the country, particularly in Kebbi, Yobe, and Zamfara. The health and program managers should concentrate malaria control resources in high‐risk states such as Kebbi, Yobe, and Zamfara rather than allocating resources uniformly across Nigeria. They should also tailor intervention packages to the local epidemiological context, recognizing that malaria risk varies spatially.

Aridity and enhanced vegetation index were confirmed as significant predictors of malaria risk in this population of children. Since aridity and the Enhanced Vegetation Index were significant predictors, the program managers should incorporate environmental and climatic information into malaria early warning systems and intervention planning. Bayesian geospatial modeling improved the precision of the prevalence estimates and showed that the addition of covariates considerably improved the explanatory ability of the model and our findings can be used as an effective tool in the identification of communities that urgently require targeted interventions by program managers and implementers; this is critical as part of an overall strategy in reducing the malaria burden given the limited public health resources available in Nigeria. Bayesian risk maps should be integrated into routine malaria surveillance and program planning to identify priority districts and monitor changes over time.

The modelling approach employed in this study can also promote effective and sustainable malaria control, prevention and eliminations efforts in this population of children in Nigeria and other similar countries. Despite ongoing national efforts, the persistence of high‐prevalence areas indicates that malaria control strategies need to be more geographically targeted and context‐specific to effectively reduce the disease burden in this vulnerable population. The study recommends additional research like a qualitative study to unearth reasons why some children living in some parts of Nigeria are more at risks of malaria compared to their counterparts living in other parts of the country.

## Author Contributions

**Justice Moses K. Aheto:** conceptualization, investigation, methodology, validation, software, data curation, formal analysis, supervision, project administration, visualization, writing – original draft, writing – review and editing, funding acquisition. **Bakare Emmanuel Afolabi:** conceptualization, funding acquisition, investigation, project administration, supervision, writing – original draft, writing – review and editing, data curation. **Dolapo O. Oniyelu:** investigation, writing – review and editing. **Deborah O. Daniel:** investigation, writing – review and editing. **Steven I. Ikediashi:** investigation, writing – review and editing. **Oluwaseun A. Mogbojuri:** investigation, writing – review and editing. **Ronke D. Olorunfemi:** investigation, writing – review and editing. **Sodiq A. Orogun:** investigation, writing – review and editing. **Afeez Abidemi:** investigation, writing – review and editing. **Idowu I. Olasupo:** investigation, writing – review and editing. **Samuel A. Osikoya:** investigation, writing – review and editing. **Samuel A. Adeyemi:** investigation, writing – review and editing. **Hapiness O. Ismail:** investigation, writing – review and editing. **Isaac Bakare:** investigation, writing – review and editing. **Samson O. Olagbami:** investigation, writing – review and editing. **Dolapo A. Bakare:** investigation, writing – review and editing. **Baiyeri Samuel:** investigation, writing – review and editing. **Temitayo V. Irewole:** investigation, writing – review and editing. **Oluwafunmilayo O. Olapade:** investigation, writing – review and editing. **Oluwafolakemi Odunola:** investigation, writing – review and editing. **Oghenekevwe R. Ajewole:** investigation, writing – review and editing. **Joshua P. Ojo:** investigation, writing – review and editing. **Segun Oyedeji:** investigation, writing – review and editing. **Wisdom Takramah:** investigation, writing – review and editing.

## Conflicts of Interest

Justice Moses K. Aheto is an Editorial Board member of Health Science Reports and the lead author of this article. He was excluded from editorial decision‐making related to the acceptance of this article for publication in the journal. All other authors declare that they have no conflict of interest.

## Transparency Statement

I, Justice Moses K. Aheto, affirms that this manuscript is an honest, accurate, and transparent account of the study being reported; that no important aspects of the study have been omitted; and that any discrepancies from the study as planned (and, if relevant, registered) have been explained.

## Supporting information


**Appendix Figure S1:** Interactive web‐based map version for Figure 2 in the main manuscript.

## Data Availability

The data that support the findings of this study are openly available in DHS Program at http://dhsprogram.com/data/available-datasets.cfm. The datasets generated and/or analyzed during the current study are available from the MEASURE DHS Program website http://dhsprogram.com/data/available-datasets.cfm.
